# High-Quality Draft Genome Sequence of the Microcolonial Black Fungus Aeminium ludgeri DSM 106916

**DOI:** 10.1128/MRA.00202-19

**Published:** 2019-04-04

**Authors:** João Trovão, Igor Tiago, Fabiana Soares, Diana Sofia Paiva, Nuno Mesquita, Catarina Coelho, Lídia Catarino, Francisco Gil, António Portugal

**Affiliations:** a Centre for Functional Ecology–Science for People & the Planet, Life Sciences Department, University of Coimbra, Coimbra, Portugal; b Fitolab–Laboratory for Plant Health, Instituto Pedro Nunes, Coimbra, Portugal; c Geosciences Center, Earth Sciences Department, University of Coimbra, Coimbra, Portugal; d CFisUC–Center for Physics of the University of Coimbra, Coimbra, Portugal; Broad Institute of MIT and Harvard University

## Abstract

Aeminium ludgeri is an extremotolerant microcolonial black fungus isolated from a biodeteriorated limestone art piece in the Old Cathedral of Coimbra, Portugal (a UNESCO World Heritage Site). The high-quality draft genome sequence of *Aeminium ludgeri* presented here represents the first sequenced genome for both the recently described fungal family *Aeminiaceae* and the genus *Aeminium*.

## ANNOUNCEMENT

Microcolonial black fungi are a diverse group of slow-growing fungi that pose three major problems when they colonize historical stone monuments, esthetic, biophysical, and biochemical biodeterioration (1–3). Microcolonial black fungi usually display high resistance to various extreme environmental conditions, being considered one of most resistant groups of eukaryotic organisms and a serious challenge in the field of biodeterioration of cultural heritage materials (4). Recently, the family Aeminiaceae (Capnodiales) was described based on the typification of the species *Aeminium ludgeri* and the genus Aeminium. *Aeminium ludgeri* DSM 106916 (strain E14) was isolated from a biodeteriorated limestone art piece in the Old Cathedral of Coimbra, Portugal, and it is characterized by slow growth, late melanization, and extremotolerance (halotolerance, xerophilia, and facultative alkaliphilia) (5). In this study, we were able to produce a high-quality draft genome sequence of *Aeminium ludgeri* DSM 106916 that constitutes valuable data for genomic studies regarding the extremotolerance pathways of microcolonial black fungi and to further understand their contribution to biodeterioration of stone monuments.

Fresh cultures of Aeminium ludgeri DSM 106916 were grown in potato dextrose agar (Difco, USA) and incubated aerobically in the dark at room temperature (28 ± 1°C), until full melanization of the cultures could be observed (6 months). Genomic DNA was extracted with a DNeasy PowerSoil extraction kit (Qiagen), and 200 ng of high-quality genomic DNA was used for DNA library preparation with the TruSeq Nano DNA library kit (Illumina, USA) and sequenced using paired-end (PE) 2 × 150-bp technology on the NextSeq 550 Illumina platform at Genoinseq (Cantanhede, Portugal). All procedures were performed according to the manufacturers’ protocols.

Sequenced reads were demultiplexed using Bcl2fastq v.2.20 (Illumina, USA) and quality filtered with Trimmomatic v.0.30 (6) using the following parameters: ILLUMINACLIP, TruSeq3-PE.fa:2:30:10, SLIDINGWINDOW:5:25, and MINLEN:50. High-quality, adapter-free reads were assembled with dipSPAdes v.3.11.1 (7) with the parameters –careful and –cov-cutoff auto. Assembled scaffolds with a size of <1,000 bp were removed from the assemblies. Assembly metrics were calculated with Quast v.4.6.1 (8). Coding gene predictions were performed with AUGUSTUS v.2.5.5 (9) with the option –especies=Botrytis cinerea for training, rRNA genes were detected using Barrnap v.0.8 (https://github.com/tseemann/barrnap), and tRNA genes were identified with ARAGORN v.1.2 (10). Coding gene annotation was carried out with DIAMOND v.0.9.22 (11) against the Swiss-Prot database (12) and with HMMER 3.1b2 (13) against the High-quality Automated and Manual Annotation of Proteins (HAMAP) (14), TIGRFAMs (15), and Pfam (16) repositories and used to determine the existing pathways using the Kyoto Encyclopedia of Genes and Genomes (KEGG) (17).

This analysis produced 43,665,202 paired reads with an average length of 150 bases for each pair (400× sequencing depth coverage and 58.57% average G+C content). Trimming of low-quality bases and removal of reads shorter than 50 bp yielded 34,772,376 high-quality paired-end reads and 5,971,206 unpaired reads. The *de novo* read assembly produced 228 scaffolds, and the longest scaffold had 704,538 bases. In total, 8,128 genes with 8,103 coding genes were identified, along with 25 RNA genes, including 23 tRNA genes and 2 rRNA genes.

### Data availability.

The raw reads and draft genome sequence of Aeminium ludgeri DSM 106916 have been deposited in the NCBI Sequence Read Archive (SRA) and GenBank databases under the accession numbers SRR8580644 and SGQK00000000, respectively, and BioProject number PRJNA520871.
